# Investigating pathways for predisposing, enabling and need factors in predicting the use of STI/HIV-testing services among Syrian and Iraqi migrants in Scania, Sweden – a cross-sectional study with directed acyclic graphs for modelling pathways to testing

**DOI:** 10.1186/s12889-022-14615-6

**Published:** 2022-11-25

**Authors:** Pia Svensson, Anette Agardh, Slobodan Zdravkovic, Benedict Oppong Asamoah

**Affiliations:** 1 Social Medicine and Global Health, Department of Clinical Sciences, Lund University, Malmö, Sweden; 2 Department of Care Science, Faculty of Health and Society and Malmö Institute for Studies of Migration Diversity and Welfare (MIM), Malmö University, Malmö, Sweden

**Keywords:** Migrant, STI/HIV-testing, Prevention, Health care utilization, Sexual risks, Directed acyclic graph

## Abstract

**Background:**

Some groups of migrants have increased vulnerability to Sexually Transmitted Infections (STI) and Human Immunodeficiency Virus (HIV) transmission partly due to a lower uptake of disease preventive activities targeting the general population in receiving country. Limited access to economic and social resources and poor language skills may exacerbate exposure to sexual risks and utilization of health services.

**Aim:**

To explore general and migrant specific predictors for STI/HIV-testing among Syrian and Iraqi migrants in Sweden and to investigate potential pathways that link predisposing, enabling and need- factors to STI/HIV-testing.

**Method:**

Cross-sectional study design based on a migration specific framework for health care utilization. Directed acyclic graphs (DAGs) were used to model assumptions about factors associated with the uptake of STI/HIV-testing services. Bi-variable and multivariable logistic regression analyses assessed individual predictors while adjusting for covariates. The magnitude of the indirect effect of mediating variables were estimated with bootstrap analyses and a method for decomposing the total effect.

**Result:**

The pathways between younger age, unmarried, and self-identifying as bi- or homosexual and testing were mainly indirect, mediated by experiences of sexual coercion and other risk behaviours. One third of the indirect mediating effect of the pathway between higher education and testing could be attributed to Swedish language skills.

**Conclusion:**

Utilization of STI/HIV-testing services among Syrian and Iraqi migrants seemed to be motivated by sexual risk exposure and risk awareness. Interventions should focus on language-adapted information about available screening services and where to go for advice on sexual wellbeing and sexual rights. Such activities should be implemented within an integration promoting framework, addressing structures that increase STI/HIV risk exposure, specifically targeting vulnerable subgroups of migrants.

## Introduction

During 2015–2016, Sweden was one of the countries in the European Union (EU) with the largest reception of asylum seekers relative to population size [1] with more than 160,000 asylum applications [2]. In 2020, 19.7% of the Swedish population of 10.3 million were foreign-born [2]. The largest migration groups as of today originate from Syria and Iraq, representing 1.8 and 1.4% of the total population, respectively [3].

Sexually transmitted infections (STI) are spread principally by sexual contact, including vaginal, anal, and oral sex [4]. Human immunodeficiency virus (HIV) is a chronic condition, however, if a person has access to well-adjusted treatment the virus loads get so low that it can no longer be transmitted [5]. About 8000 persons currently live with HIV in Sweden [6]. However, cases may be underreported as most HIV infections originate from individuals who are undiagnosed and unaware of their infection [7]. In 2018, foreign born represented 75% of all new HIV cases (out of 4.7 per 100,000 inhabitants), including diagnosed and undiagnosed cases before migrating [8]. Foreign born persons are also overrepresented with regards to the incidence of new cases that have been transmitted in Sweden [9]. Migrants originating from HIV endemic countries are therefore a key population for HIV-prevention in recipient countries in Europe [10]. The adult HIV prevalence in the middle east and north Africa regions (MENA) is the lowest in the world (0.1%). However, over the past two decades the region has had the highest increase in the world [11]. Between 2010 and 2019, Iraq had an annual increase in HIV cases of 14.7%, which can partly be explained by streamlining screening and reporting [12]. Conflicts and war in Syria has contibuted to heightened vulnerability to HIV transmission and reduced access to services [13]. Since the migration situation increase the risks for STI/HIV, researchers have argued that preventive activities must include migrants also from low HIV prevalence countries [14]. STI/HIV-testing is a biomedical primary prevention for those exposed to risks and a core activity for preventing further transmission, and for facilitating access to treatment and care for those diagnosed [15, 16]. Studies have found lower HIV-testing uptake [17] and late presentation [18] among different migrant populations in Europe, which counteract the possibilities for early detection. The migration situation is characterized by practical challenges, economic instability, and limited access to social networks that can provide information about available services or assist with life struggles [19, 20, 21]. Language difficulties, discrimination, and low trust in health care providers further constrain access to health care [20, 22, 23]. A recent report from the Equality Ombudsman (DO) stated that foreign born persons were twice as likely than Swedish born persons to refrain from seeking health care on ground of discrimination, despite need. Bi- or homosexuals also abstained from seeking care to a larger extent [24]. Moreover, migrant legal status and awareness of rights are vital for access to health services [19, 20, 22]. A Swedish study found that fear of deportation was the most cited reason for avoiding HIV-screening among newly arrived migrants [14]. The susceptibility for transmission also increases during the migration process [25]. Poor socioeconomic conditions and marginalisation enhance sexual risk exposure for STI/HIV including related risk behaviours such as substance use and alcohol consumption [25]. Such factors reduce access to services and undermine people’s ability to act on prevention advice [17]. Uncertainties related to waiting for decisions on asylum application have been identified as causing vulnerabilities [26]. 

Yang and Hwang [19] developed a framework specifically adapted to understand migrants Heath Service Utilization (HSU). The framework conceptualizes predictors on three levels corresponding to predisposing, enabling, and need-related factors and theorizes about mediating and intervening relationship between general and migrant specific factors on different levels. Predisposing predictors refers to sociodemographic characteristics, health beliefs, and migration legal status (e.g., undocumented, asylum seeker, permitted residence) [19]. Level of education is a strong predictor for the uptake of STI/HIV-testing on the predisposing level. Women and bi-or homosexual persons are also more likely to get tested, which has been explained by the availability of services related to reproductive care, and targeted interventions [20, 23, 27]. Enabling factors, according to the framework, refer to resources that may facilitate access to health care, such as social and economic assets, language skills and trust in health care. Need level factors encompass both clinically assessed health needs and self-perceived needs [19]. For example, low risk perception and not feeling sick has been identified as barriers to STI/HIV-testing and determinants for late presentation [20]. Understanding the processes that underlie use of HIV-testing is crucial for designing services and targeted interventions [28]. The aim of this study was to investigate general and migrant specific predictors of STI/HIV-testing among migrants from Syria and Iraq who have resettled in Scania, Sweden. Potential pathways for predisposing, enabling and need level factors for the prediction of STI/HIV-testing were explored based on a migrant-specific framework for HSU and directed acyclic graphs (DAGs). We aimed to answer the following research questions, 1) what general and migrant specific factors predict STI/HIV-testing among migrants from Syria and Iraq, who have recently resettled in Scania, and 2) what potential pathways link the predisposing, enabling, and need level factors for the prediction of STI/HIV-testing?

## Method

### Study setting

In 2020, Scania, a region in south of Sweden, had a population of 1.38 million inhabitants whereof 23% was foreign-born [29]. Syria and Iraq were the most common countries of origin, accounting for 2.1 and 1.8% of the total population, respectively [3]. In Sweden, STI/HIV-testing and treatment is organized in accordance with the Communicable Diseases Act (2004:168) [30]. Early detection is a principal goal of the National strategy against HIV/STI and other infectious diseases [31]. Accordingly, testing is available free of charge at health care centres, infection clinics, youth clinics, and by some Non-Governmental Organisations (NGOs) [32]. To increase the possibilities to detect an ongoing HIV-infection at an early stage, HIV-test is offered in connection to the health assessment, according to Act (2008: 344) on healthcare for asylum seekers, etc. [33]. Upon reception of residence permit, newly arrived refugees and other migrants in Scania are offered information about the health system, sexual health and STI/HIV as part of the civic orientation under the Establishment Act (2010:197) for newly arrived immigrants [34].

### Participants

The survey was limited to persons from Syria and Iraq residing in Scania, who came to Sweden as refugees and who received residence permit during the period September 1st, 2012, and August 31st, 2016. Respondents were between 20 and 64 years old upon reception of their residence permits.

### Data collection

This cross-sectional study is based on data from MILSA 2.5, a comprehensive survey conducted as part of MILSA, a research platform for migration and health, led jointly by Malmö University and the County Administrative Board of Scania. The overall purpose of the survey was to investigate health and health related factors among newly arrived adult migrants in Scania, who had completed the establishment phase (approximately 2 years). MILSA 2.5 cover questions on various health-related aspects such as sexual health, health service utilization, living conditions, and social relations. Questions specific for the migration situation included waiting time for residence permit, language skills and participation in activities related to the establishment plan. The survey was administered during fall 2018 in collaboration with the Swedish Employment Service, Region Scania, Malmö municipality, Lund University and Uppsala University [35]. The selection of respondents was conducted through a selection framework that defined, identified, and enabled connection to the individuals in the population. The framework was created with data from the register of the total population (RTB) (*n* = 11,286 persons). The survey was distributed in Arabic to a randomly selected unstratified sample of 10,000 persons from Syria and Iraq with addresses in Scania. Five mailings were sent out between September–December 2018, whereof the first was administered online only and the others were sent out as paper questionnaires but with the possibility to be completed online using a personal login. In total 3226 respondents returned the survey (32.3%). Out of the non-respondents, four informed that they refused to answer, 36 were returned due to change of address and 45 surveys were returned with no given reason. The rest did not reply. Data was weighted to adjust for skewness in the non-responses by calibrating the results with register data. To mitigate the effect of selection bias, data was weighted according to population distribution of gender, age, birth country, income, and municipality [35]. Selection, data collection and compilation of data, retirement of register data, and calibration of weights was completed by Enkätfabriken AB (www.enkatfabriken.se) and Statistics Sweden (www.SCB.se).

## Measures

### Dependent variable

The dependent variable was “STI/HIV-testing in the past 12 months”, dichotomized as “no” or “yes”, coded as 0 and 1. The option “don’t remember” was re-coded as missing.

### Covariates

Covariates were organized based on the migrant-specific framework for HSU [19]. We categorized “waiting time for residence permit”, “language skills”, and “trust in health care” as migrant specific variables, although, the meaning of general factors should be understood in migration context. We expected that “waiting time for residence permit” would influence testing behaviour in the way that shorter waiting time predicted greater likelihood of getting tested. Further, that following factors would be associated with STI/HIV-testing on a predisposing level—sex, age, sexual orientation, marital status, and education. The “age” variable was created from a continuous scale and categorized in three age groups 18–34, 35–44, and 45 years or older. Sexual orientation was indicated by the question: “What do you consider yourself today …?” with the alternative’s heterosexual, bisexual, homosexual, or other. In the analysis bisexual and homosexual were grouped as one category. Marital status was categorized as “married”, “unmarried”, “divorced” or “widow/ widower”, whereby the two latter were merged. Education was measured as years of schooling and categorized as “pre−/high school” (0–9 years), “upper secondary school” (10–12 years) or “post-secondary” (13+ years and university degree). We hypothesized that communication skills in the native language of the receiving country, access to social support, financial stability, and trust in the health care and trust in interpreters, would act as enablers to HIV/STI testing. “Financial difficulties ” was measured by how often the respondents had had difficulties paying their bills in the past 12 months—"every month”, “half of the months”, “occasionally”, or “never”. In the analysis the two former categories were collapsed to indicate “often”. Trust in the health care and in interpreters was dichotomized as “no” and “yes” by merging “great” and “pretty good “as yes, and “not particularly good” and “not at all” as no. “No opinion” was coded as missing. Emotional and practical social support were indicated by the questions *“*Do you feel that you have someone who can give you proper personal support to cope with life stressors and problems” and “Can you get help from someone if you are sick or have practical problems, such as borrowing things, get help with reparations, receive information and advice?”, respectively. Both were dichotomized as “no” or “yes”. Since we do not have data on actual need, e.g., symptoms or known exposure, sexual risk exposure, and risk behaviours associated with sexual risks, such as alcohol consumption and substance use, were used as proxies to need. Sexual risk was measured with two questions, “have you been sexually harassed in the past 12 months”, and “it happens that people are drawn into sexual acts without wanting to. Have you ever without wanting to, been forced into such an act? Both were re-coded and dichotomized from “no”, “yes”, “one time” and “yes, several times”, to “yes” and “no”. Substance use the past 12 months was dichotomized as never and one time or more. Alcohol consumption was indicated by consumption the past 12 months: “never”, “1 time/month” or “less, 2-4 times/month”, “2–3 times/week”, or “four times /week or more”. This variable was kept as three categories: “never”, “1–4 times/month” and “two times or more a week”.

### Directed Acyclic Graph (DAG)

**Fig. 1:**
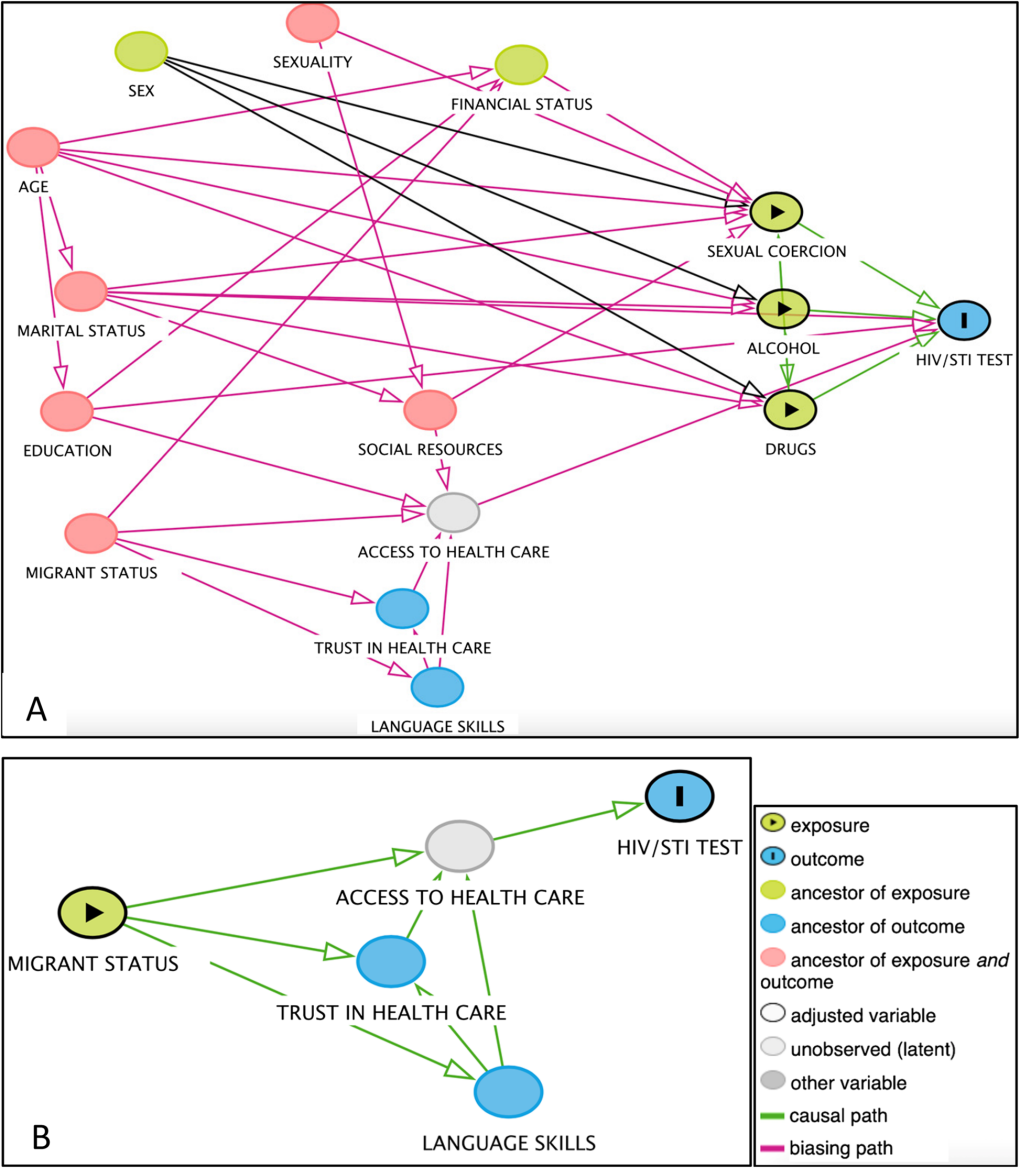
Directed acyclic graph illustrating assumptions about predictor- outcome relationship for the outcome STI/HIV-testing. **A** = full pathway model, **B** = “migration specific” pathway (developed from www.dagitty.net using DAGitty version 3.0)

We conducted a DAG to illustrate direct and mediated pathways between predictors on predisposing, enabling, need/risk levels, and the outcome. A DAG is a useful tool for mapping assumptions about casual pathways between predictor variables and an outcome and identify the presence of confounding for the predictor-outcome relationship, e.g., backdoors that needs to be blocked [36, 37]. We modelled potential pathways to STI/HIV-testing. Predictors could therefore be both exposures and confounders. Covariates on the path could mediate or modify the predictor-outcome relationship. As illustrated in the DAG (Fig. 1a), we assumed that there would be a direct pathway (association) between “education” (predisposing level) and STI/HIV-testing. Further, there would be three independent indirect pathways between “age” and “marital status” and “sexual orientation” and STI/HIV-testing, via “sexual coercion”. To investigate the total effect of this pathway according to the DAG, the backdoors “social support” and “financial situation” needed to be blocked. Marital status was also assumed to be associated with the outcome, based on the assumption that unmarried marital status enhances the probability of engaging in risk-taking behaviours. The green lines from the exposure variables to the outcome indicate direct pathways, they have been substituted by proxy variables, by increasing the risk of actual needs. Access includes the physical availability of testing services and the perceived access [19]. “Language” and “trust in health care” were used as proxy variables for “access to health care” (unobserved) and was assumed to mediate the pathway between waiting time for residence permit and the outcome (Fig. 1b).

## Analyses

Variables were controlled for multicollinearity using a correlation matrix and verified with variant inflation factor (VIF) set at 5. Multicollinearity was not detected, but the collinearity diagnostics indicated interdependence between “sexual harassment” and “sexual coercion”. Goodness of fit between independent variables and the outcome was assessed with Pearson Chi square. Bivariate logistic regression analysis was conducted to investigate independent predictors on HIV/STI testing. Statistical significance was set at two-tailed α of 0.05, with 95% confidence intervals (CI). Variables with *p*-value ≤0.2 were included in the multivariable logistic regression models [38]. At this step “sexual harassment” was omitted to avoid overadjustments. The DAGs were predominately used for illustrating potential pathways between the different levels of variables. The pathways were further examined with regression analyses whereby the strength of association between predisposing predictor variables and the outcome was estimated by controlling for biased pathways. To examine the influence of enabling and need/risk proxy variables, one main multivariable regression analysis was carried out by entering variables stepwise through three blocks of predisposing, enabling, and need/risk variables. The fully adjusted model consisted of all variables. Data analyses were carried out in IBM SPSS Statistics® version 25.0. The DAG and results from the regression analysis informed further analyses on the indirect effect of mediators on the hypothesized pathways (associations) between predisposing variables and outcome. These were estimated with bootstrap analyses and method for decomposing the total effect into direct and indirect effects of association, as suggested by Buis [39] using Stata/SE 17.0 for Mac. This method provided a way for categorical variables to have any distribution [39]. We estimated the proportion of the total effect of the relationship between marital status and the outcome that could be attributed to access to social support, financial situation, alcohol consumption and exposure to sexual coercion. Additional analyses were conducted to examine the proportion of the pathway between sexuality and the outcome that could be explained by access to social support and exposure to sexual coercion. Finally, we investigated the influence of language on the pathway between education and testing, as language is a known barrier/facilitator to access to health care, and we assumed that there was a relationship between education and language skills. The “migrant specific” pathway illustrated in the DAG was examined by analysing the mediating effect of language skills and trust in health care (proxies for access) on the relationship between waiting time for residence permit and STI/HIV-testing (Fig. 1b).

## Results

A total of 1234 (38.3%) women and 1992 (62.7%) men participated in the study. Most respondents (42.2%) were between 18 and 34 years old (mean and median 38 and 36 years, respectively). The majority were married (74.1%) and defined themselves as heterosexuals (90.5%). Almost 4 % stated their sexual orientation as bi- or homosexual. Education level was equally distributed over men and women, 41.4% had 13 or more years of schooling, and 35% had lower levels of education. Most of the respondents had received residence permit within 6 months after migration to Sweden (42%), while 7.5% had waited more than 18 months for their residence permit. With regards to the enabling variables, 28% of the study sample reported that they had experienced financial difficulties during the past 12 months. Half of the respondents never had these problems. The majority could communicate well or make themselves understood in the Swedish language (52.2 and 34.8%, respectively), while 13% reported that they could not speak the language. About 50% revealed that they trusted the health care and interpreters, while the other half had low or no trust in both health care and interpreters. More than half of the respondents reported access to emotional social support (57.7%) and the majority (85.2%) had access to practical social support. Regarding the need/risk variables*,* almost 2% had experienced sexual harassment the past 12 months (2.2 and 1.7% for women and men, respectively). Three percent had been exposed to sexual coercion (2.6 and 3.3% for women and men, respectively). Two percent had used drugs in the past 12 months, and slightly more (2.5%) consumed alcohol more than two times weekly. Four percent of those surveyed had been tested for STI/HIV in the past 12 months, more men than women had done so (4.5 and 3.1%, for men and women respectively) (Table 1).

**Table 1:** Frequency of outcome variable, predisposing, enabling and need/risk variables. MILSA 2.5 data on migrants from Syria and Iraq residing in Scania, Sweden who received residence permit between 2012 and 2016 (*n* = 3226)

Variable	All, % (N) (***N*** = 3226)	Women, % (N) (38.3%, ***N*** = 1234)	Men, % (N) (62.7%, ***N*** = 1992)
***Outcome***			
HIV/STI testing last 12 months	4.0% (126/3159)	3.1% (38/1203)	4.5% (88/1956)
Don’t remember	5.3% (167/3159)	5.6% (68/1203)	5.1% (100/1956)
*(Missing)*	*2.1% (67)*	*2.5%* (31)	*1.8%* (36)
***EXPLANATORY VARIABLES***			
***Predisposing***			
**Age**			
18–34	42.0% (1356/3226)	43.9% (541/1234)	40.9% (815/1992)
35–44	31.8% (1027/3226)	31.6% (390/1234)	32.0% (637/1992)
45+	26.1% (843/3226)	24.5% (303/1234)	27.1% (541/1992)
**Marital status**			
Married	74.1% (2369/3199)	82.5% (1010/1225)	68.8% (1359/1974)
Unmarried	17.8% (570/3199)	5.4% (66/1225)	25.6% (504/1974)
Divorced/widow	8.1% (2593199)	12.1% (149/1225)	5.6% (111/1974)
*(Missing)*	*0.8%* (27)	*0.7%* (9)	*0.9%* (18)
**Sexual orientation**			
Heterosexual	90.5% (2370/2620)	87.8% (800/911)	91.8% (1570/1710)
Bi/homosexual	3.8% (101/2620)	4.2% (38/911)	3.6% (62/1710)
Other	5.7% (150/2520)	8.0% (73/911)	4.5% (77/1710)
*(Missing)*	*18.8% (606)*	26.2% (323)	14.2% (282)
**Education**			
0–9 years	35.0% (1119/3194)	36.6% (448/1223)	34.0% (671/1971)
10–12 years	23.6% (752/3194)	24.4% (299/1223)	23.0% (453/1971)
13+ years	41.4% (1323/3194)	38.9% (476/1223)	43.0% (847/1971)
*(Missing)*	*1.0%* (32)	*0.9%* (11)	*1.0%* (21)
**Time waiting for residence permit**			
0–6 months	42% (1334/3173)	48.3% (587/1215)	38.1% (747(1958)
7–12 months	37% (1175/3173)	30.9% (376/1215)	40.8% (800/1958)
13–18 months	13.4% (426/3173)	12.8% (155/1215)	13.9% (271/1958)
> 18 months	7.5% (238/3173)	8.0% (98/1215)	7.1% (140/1958)
*(Missing)*	1.6% (53)	*1.5%* (19)	*1.7%* (34)
***Enabling variables***			
**Financial difficulties**			
Often	28.3% (892/3158)	25.8% (308/1195)	29.8% (584/1963)
Occasionally	19.3% (611/3158)	17.6% (210/1195)	20.4% (401/1963)
Never	52.4% (1655/3158)	56.7% (677/1195)	49.8% (978/1963)
**Language**		*3.2%* (39)	*1.5%* (29)
Can communicate	52.2% (1647/3155)	48,3% (583/1208)	54.6% (1063/1947)
Can make myself understood	34.8% (1098/3155)	35.3% (427/1208)	34.5% (671/1947)
Cannot speak Swedish	13.0% (410/3155)	16.4% (198/1208)	10.9% (212/1947)
*(Missing)*	2.2% (71)	2.1% (26)	2.3% (46)
**Social support (emotional)**	57.7% (1840/3186)	64.5% (785/1216)	53.5% (1055/1970)
*(Missing)*	1.2% (40)	1.5% (18)	1.1% (22)
**Social support (practical)**	85.2% (2719/3191)	88.7% (1081/1218)	83.1% (1638/1973)
*(Missing)*	1.1% (35)	1.3 (16)	1.0% (19)
**Trust health care**	52.2% (1571/3007)	52.5% (613/1169)	52.1% (957/1838)
*(Missing)*	*6.8% (219)*	5.2% (65)	7.7% (154/1992)
**Trust interpreters**	48.6% (1311/2696)	42.9% (517/1203)	40.8% (794/1944)
*(Missing)*	*16.4 (530)*	*2.5%* (31)	*2.5 (49)*
***Need/risk variables***			
**Sexual harassment**	1.9% (60/3145)	2.2% (27/1197)	1.7% (34/1948)
*(Missing)*	2.5% (81)	*3.0%* (37)	*2.2%* (45)
**Sexual coercion**	3.0% (93/3067)	2.6% (30/1156)	3.3% (63/1911)
*(Missing)*	4.9% (159)	*6.3% (78)*	*4.1% (81)*
**Drug use**	2% (64/3127)	0.6% (7/1187)	2.9% (57/1940)
*(Missing)*	3.1% (99)	3.8% (48)	2.6% (53)
**Alcohol consumption**			
Never	83.6% (2658/3179)	92.2% (1119/1214)	78.3% (1539/1966)
≥ 2 times/week	2.5% (79/3179)	1.2% (14/1214)	3.3% (64/1966)
1–4 times/month	13.9% (443/3179)	6.7% (81/1214)	18.4% (362/1966)
*(Missing)*	*1.4%* (48)	*1.7% (20/1234)*	*1.3%* (26)

Percentages and frequencies of included variables. Total and distributed by gender

### Unadjusted effect of predisposing, enabling and need/risk variables on STI/HIV-testing

The Chi^2^ analyses showed significant relationship between marital status, sexuality, education, language, lack of trust in health care, practical social support, all the need/risk factors and the outcome (Table 2).

**Table 2:** Pearson’s chi^2^ test of association between independent variables and outcome STI/HIV-testing. MILSA 2.5 data on migrants from Syria and Iraq residing in Scania, Sweden who received residence permit between 2012 and 2016 (*n* = 3226)

		HIV/STI testing			
		Yes	No	Total	Chi^**2**^***P***-value
***PREDISPOSING***					
**Sex**	Male	88 (4.7%)	1768 (95.2%)	1856	.065
	Female	38 (3.3%)	1098 (96.6%)	1136	
	**Total**	**126 (4.2%)**	**2866 (95.7%)**	**2992 (92.7%)**	
**Age**	18–34 years	77 (6.0%)	1202 (93.9%)	1279	.000^**^
	35–44 years	34 (3.5%)	911 (96.4%)	945	
	45+ years	14 (1.8%)	752 (98.1%)	766	
	**Total**	**125 (4.2%)**	**2865 (95.8%)**	**2990 (92.6%)**	
**Marital status**	Married	62 (2.8%)	2142 (97.1%)	2204	.000^**^
	Unmarried	55 (10.2%)	479 (89.7%)	534	
	Divorced/widow	8 (3.4%)	222 (96.5%)	230	
	**Total**	**125 (4.2%)**	**2843 (95.7%)**	**2968 (92.0%)**	
**Sexual orientation**	Heterosexual	95 (4.2%)	2152 (95.7%)	2247	.002^*^
	Bi/homosexual	11 (11.7%)	83 (88.2%)	94	
	Other	4 (2.9%)	132 (97.0%)	136	
	**Total**	**110 (4.4%)**	**2367 (95.5%)**	**2477 (76.7%)**	
**Education**	0–9 years	26 (2.5%)	986 (97.4%)	1012	.000^**^
	10–12 years	20 (2.8%)	685 (97.1%)	705	
	13+ years	77 (6.1%)	1172 (93.8%)	1249	
	**Total**	**123 (4.1%)**	**2843 (95.8%)**	**2966 (91.9%)**	
**Time waiting for residence permit**	0–6 months	41 (3.2%)	1204 (96.7%)	1245	.066
	7–12 months	48 (4.3%)	1047 (95.6%)	1095	
	13–18 months	19 (4.9%)	368 (95.0%)	387	
	18+ months	15 (6.9%)	201 (93.0%)	216	
	**Total**	**123** (4.1%)	**2820** (95.8%)	**2943 (91.2%)**	
***ENABLING VARIABLES***					
**Financial difficulties**	Often	43 (5.2%)	771 (94.7%)	814	.146
	Occasionally	24 (4.2%)	543 (95.7%)	567	
	Never	56 (3.5%)	1506 (96.4%)	1562	
	**Total**	**123**(4.1%)	**2820 (95.8%)**	**2943 (91.2)**	
**Language**	Can communicate	84 (5.4%)	1471 (94.5%)	1555	.003^*^
	Can manage	28 (2.7%)	982 (97.2%)	1010	
	Cannot speak	11 (3.0%)	354 (96.9%)	365	
	**Total**	**123** (4.1%)	**2807** (95.8%)	**2930 (90.8)**	
**Trust health care**	Yes	46 (3.1%)	1406 (96.8%)	1452	.038^*^
	No	63 (4.6%)	1281 (95.3%)	1344	
	**Total**	**109** (3.9%)	**2687** (96.1%)	**2796 (86.6%)**	
**Trust interpreters**	Yes	45 (3.7%)	1156 (96.2%)	1201	.430
	No	57 (4.3%)	1247 (95.6%)	1304	
	**Total**	**102** (4.0%)	**2403** (95.9%)	**2505 (77.6%)**	
**Social support (emotional)**	Yes	64 (3.7%)	1652 (96.2%)	1716	.121
	No	61 (4.8%)	1187 (95.1%)	1248	
	**Total**	**125** (4.2%)	2839 (95.7%)	**2964 (91.8%)**	
**Social support (practical)**	Yes	98 (3.8%)	2422 (96.1%)	2520	.022^*^
	No	28 (6.2%)	419 (93.7%)	447	
	**Total**	126 (4.2%)	2841 (95.7%)	**2967** (91.9%**)**	
***NEED/RISK***					
**Sexual harassment (past 12 months)**	Yes	12 (21.0%)	45 (78.9%)	57	
	No	112 (3.8%)	2779 (96.1%)	2891	
	**Total**	**124 (4.2%)**	**2824 (95.7%)**	**2948 (91.3%)**	
**Sexual coercion (past 12 months)**	Yes	21 (23.8%)	67 (76.1%)	88	.000^**^
	No	100 (3.5%)	2690 (96.4%)	2790	
	**Total**	**121 (4.2%)**	**2757 (95.7%)**	**2878 (89.2%)**	
**Drug use (past 12 months)**	Never	113 (3.9%)	2335 (81.9%)	2848	.000^**^
	≥1 time	12 (19.3%)	50 (80.6%)	62	
	**Total**	**125 (4.2%)**	**2785 (95.7%)**	**2910 (90.2%)**	
**Alcohol consumption (past 12 months)**	Never	79 (3.1%)	2405 (96.8%)	2484	.000^**^
	≥2 times/week	10 (13.5%)	64 (86.4%)	74	
	1–4 times/month	36 (9.0%)	364 (91.0%)	400	
	**Total**	**125 (4.2%)**	**2833 (95.7%)**	**2958 (91.6%)**	

Test for goodness of fit between independent variables: predisposing variables, enabling variables, and need/risk factors and dependent variable HIV/STI testing

^*^Significance level *p* < 0.05

^**^Significance level *p* < 0.00

The unadjusted relationship between predisposing, enabling, and need/risk factors and STI/HIV-testing are presented in Table 3. The results showed that the odds of having tested decreased with age. Respondents aged 18–34 years were three times more likely to get tested compared to the oldest age group. Testing was almost four times higher amongst unmarried compared to married persons.

Respondents self-identified as bi-or homosexuals were three times more likely to get tested compared to heterosexual respondents. Higher level of education was also associated with greater likelihood of getting testing compared to lower education levels. Respondents who had waited for residence permit for 18 months or longer was twice as likely to have tested compared to those who had waited 6 months or shorter. The bivariate analysis indicated no significant difference between men and women with regards to STI/HIV-testing.

**Table 3:** Bivariate logistic regression of the association between predisposing, enabling, and need/risk variables and STI/HIV-testing. MILSA 2.5 data on migrants from Syria and Iraq residing in Scania, Sweden who received residence permit between 2012 and 2016 (*n* = 3226)

	HIV/STI testing		
	***n***	OR	95%CI
***PREDISPOSING VARIABLES***			
**Sex**			
Female (ref)	1109	1	
Male	1892	1.44	0.98-2.13
**Age**			
18–34 years	1035	3.48	1.95–6.21**
35–44 years	967	2.04	1.08–3.83*
45+ years (ref)	1000	1	
**Marital status**			
Married (ref)	2284	1	
Unmarried	454	3.93	2.69–5.73**
Divorced/widow	237	1.29	0.62–2.71
**Sexual orientation**			
Heterosexual (ref)	2289	1	
Bi/homosexual	78	3.03	1.57–5.84**
Other	135	0.74	0.27–1.97
**Education**			
0–9 years (ref)	934	1	
10–12 years	675	1.09	0.60–1.98
13+ years	1363	2.48	1.57–3.90**
**Waiting time for residence permit**			
0–6 months (ref)	1325	1	
7–12 months	1094	1.32	0.86–2.02
13–18 months	365	1.48	0.85–2.59
18+ months	171	2.15	1.16–3.96*
***ENABLING VARIABLES***			
**Financial difficulties**			
Often (ref)	818	1	
Occasionally	567	0.80	0.48–1.33
Never	1567	0.66	0.44–1.00
**Language (Swedish)**			
Cannot speak (ref)	364	1	
Can manage	1012	0.96	0.47–1.97
Can communicate	1563	1.91	0.99–3.66
**Social support (emotional)**			
No (ref)	1286	1	
Yes	1688	0.75	0.52–1.08
**Social support (practical)**			
No (ref)	451	1	
Yes	2526	0.59	0.38–0.92*
**Trust health care**			
No (ref)	1390		
Yes	1419	0.66	0.45–0.98*
**Trust interpreters**			
No (ref)	1349		
Yes	1199	0.86	0.58–1.28
***NEED/RISK***			
**Sexual harassment (past 12 months)**			
No (ref)	2907	1	
Yes	50	6.45	3.29–12.63**
**Sexual coercion (past 12 months)**			
No (ref)	2805	1	
Yes	77	8.46	4.97–14.38**
**Drug use (past 12 months)**			
Never (ref)	2867	1	
≥ 1 time	53	5.86	3.03–11.30**
**Alcohol consumption (past 12 months)**			
Never (ref)	2521	1	
1–4 times/month	380	3.02	2.01–4.54**
≥ 2 times/week	68	4.93	2.46–9.87**

Unadjusted Odds Ratios (OR) and 95% confidence intervals (95%CI)

^*^Significance level *p* < 0.05

^**^Significance level *p* < 0.001

With regards to the enabling variables, respondents with lower access to practical social support were more likely to get tested compared with those without this support, whereas emotional social support did not seem to have a significant influence on whether one got tested or not. Similarly, those who lacked trust in the health care were more likely to have tested, while trust in interpreters did not influence testing behaviour. Language skills were only borderline significant in the direction that those who could communicate in Swedish were more likely to get tested compared to those faced with language barriers. All need/risk factors were associated with the outcome. The likelihood of having tested for STI/HIV was over six times higher among respondents with experiences of sexual harassment and eight times higher for those who had been exposed to sexual coercion compared to respondents without these experiences. Substance use and alcohol consumption also seemed to influence testing behaviour. Having used drugs at least one time the past year was associated with an almost six times greater probability of testing, and the odds of testing increased with alcohol consumption.

### Multivariable regression analyses

Assumptions illustrated in the DAG were examined through a multivariable logistic regression analysis performed in three steps. Model I adjusted for predisposing variables. Model II estimated the ‘partial’ direct effect, after adjusting for mediation by the enabling variables. The final and fully adjusted model (Model III) indicated the effect estimates of associations after adjusting for variables on all levels (Table 4). The result from Model I showed that younger age was associated with a more than two times higher likelihood of having tested for STI/HIV, compared to the oldest age-group. Those who were unmarried compared to married, bi- or homosexual compared to heterosexual, and who had high compared to low levels of education, were also more likely to have tested. Waiting time for residence permit did not influence on the probability of STI/HIV-testing after adjusting for other predisposing covariates. The result from Model II confirmed significant associations between the outcome and younger age, unmarried marital status, bi-or homosexual, and higher education. These pathways were partially mediated by the enabling variables. This effect disappeared in Model III, suggesting a strong relationship between the risk/need variables and the outcome, confirming the assumptions illustrated in the DAG. A direct pathway between education and testing persisted in Model III, showing that persons with higher levels of education were almost twice as likely to get tested compared to those with lower levels of education, regardless of access to or absence of enabling resources or exposure to risks. A direct pathway between sexual coercion and STI/HIV-testing was also confirmed. The probability of having tested was more than three times higher among those with experiences of sexual coercion, compared with having no such experience after adjusting for all predisposing and enabling variables and the other risk factors. The odds of testing also increased with alcohol consumption (Table 4).

**Table 4:** Multivariate logistic regression analysis of associations between predictor variables and STI/HIV-testing. MILSA 2.5 data on migrants from Syria and Iraq residing in Scania, Sweden who received residence permit between 2012 and 2016 (*n* = 3226)

	Model I		Model II		Model III	
	n	AOR (95%CI)	n	AOR (95%CI)	n	AOR (95%CI)
**Sex**						
Female (ref)	817		756		713	
Male	1623	1.07 (0.67–1.71)	1477	1.15 (0.70–1.91)	1407	1.10 (0.65–1.86)
**Age**						
18–34 years	883	2.55 (1.25–5.21)^*^	807	2.27 (1.09–4.71)^*^	771	1.88 (0.89–3.95)
35–44 years	801	2.14 (1.03–4.46)^*^	729	1.77 (0.83–3.77)	694	1.67 (0.78–3.58)
45+ years (ref)	756		697		655	
**Marital status**						
Married (ref)	1874		1734		1639	
Unmarried	404	2.65 (1.65–4.25)^**^	353	2.06 (1.23–3.43)^*^	342	1.52 (0.88–2.62)
Divorced/widow	162	1.64 (0.71–3.78)	146	1.90 (0.81–4.45)	139	1.43 (0.60–3.40)
**Sexuality**						
Heterosexual (ref)	2256		2053		1948	
Bi/homosexual	76	3.68 (1.83–7.41)^**^	69	3.04 (1.39–6.62)^*^	65	2.18 (0.92–5.13)
Other	129	0.91 (0.33–2.48)	111	0.60 (0.14–2.50)	107	0.58 (0.13–2.41)
**Education**						
0–9 years (ref)	675		598		556	
10–12 years	537	1.10 (0.56–2.13)	487	1.38 (0.66–2.87)	464	1.31 (0.61–2.80)
13+ years	1228	2.24 (1.32–3.80)^*^	1148	2.16 (1.14–4.09)^*^	1100	1.93 (1.00–3.72)^*^
**Waiting for residence permit**						
0–6 months	953	0.51 (0.23–1.13)	1019	0.58 (0.26–1.26)	968	0.55 (0.24–1.21)
7–12 months	762	0.56 (0.25–1.25)	812	0.58 (0.26–1.28)	772	0.57 (0.25–1.27)
13–18 months	265	0.50 (0.19–1.27)	286	0.67 (0.28–1.63)	272	0.64 (0.26–1.59)
18+ months (ref)	106		116		108	
**Financial difficulties**						
Often (ref)			620		587	
Occasionally			442	0.62 (0.33–1.17)	417	0.69 (0.32–1.24)
Never			1171	0.71 (0.44–1.15)	1116	0.83 (0.50–1.37)
**Language (Swedish)**						
Cannot speak (ref)			235		219	
Can manage			733	1.05 (0.36–3.02)	694	1.14 (0.38–3.37)
Can communicate			1265	1.70 (0.62–4.66)	655	1.77 (0.62–5.00)
**Trust health care**						
No (ref)			1121		1056	
Yes			1112	0.74 (0.48–1.16)	1064	0.77 (0.48–1.22)
**Social support (practical)**						
No (ref)			315		301	
Yes			1918	0.85 (0.48–1.16)	1819	0.92 (0.50–1.67)
**Sexual coercion (past 12 months)**						
Yes					60	
No (ref)					2060	3.75 (1.84–7.63)^**^
**Drug use (past 12 months)**						
Never (ref)					2075	
≥ 1 time					45	2.06 (0.92–4.64)
**Alcohol consumption (past 12 months)**						
Never (ref)					1767	
1–4 times/month					298	2.34 (1.41–3.91)^*^
≥ 2 times/week					55	2.67 (1.09–6.55)^*^

Adjusted Odds Ratios (AOR) and 95% confidence intervals (95%CI). Model I: Adjusted for sex, age, marital status, sexuality, education, and waiting time for residence permit; Model II: Adjusted for Model I + financial difficulties, language, trust in health care, social support; Model III: adjusted for Model I and II + sexual coercion, drug use, and alcohol consumption

*NS* not statistically significant

^*^Significance level *p* < 0.05

^**^Significance level *p* < 0.001

The result from the regression analyses suggested that the enabling variables mainly mediated the pathway between the predisposing and need/risk variables. To verify this, and to increase our understanding of the pathways to testing, we explored the relationship between predisposing and enabling variables and sexual coercion, as a risk factor for STI/HIV, by carrying out regression analyses with sexual coercion as the outcome. The reference categories for some of the variables were reversed to match the new outcome. Table 5 show the crude model and each level of variables adjusted for the same level variables. The final model (Model III) is the fully adjusted model. For simplicity, the table only display the lowest indicator value. The result showed that to be unmarried, bi- or homosexual, having financial difficulties, and low access to social support, were associated with almost two to three times higher probability of exposure to sexual coercion (Table 5).

**Table 5:** Crude and adjusted odds ratio for the association between covariates and sexual coercion. MILSA 2.5 data on migrants from Syria and Iraq residing in Scania, Sweden who received residence permit between 2012 and 2016 (*n* = 3226)

Experience of sexual coercion	Crude model		Model I^**a**^ predisposing		Model II^**b**^ enabling		Model III^**c**^ fully adjusted	
	OR	(95%CI)	AOR	(95%CI)	AOR	(95%CI)	AOR	(95%CI)
***PREDISPOSING***								
Sex (female)	1.28	(0.82–2.00)	0.84	(0.50–1.42)			0.80	(0.47–1.39)
Age 18–34 years	2.65	(1.45–4.85)^*^	1.67	(0.82–3.40)			1.79	(0.86–3.74)
Unmarried	3.93	(2.52–6.13)^**^	3.04	(1.75–5.27)^**^			2.70	(1.53–4.76)^*^
Bi/homosexual	2.58	(1.17–5.70)^*^	2.94	(1.29–6.69)^*^			2.51	(1.05–5.99)^*^
Lower education	0.50	(0.30–0.83)^*^	0.61	(0.34–1.08)			0.62	(0.33–1.15)
Longer waiting time for residence permit	2.68	(1.35–5.30)^*^	1.08	(0.53–2.20)			1.77	(0.76–4.12)
***ENABLING***								
Financial difficulties	2.40	(1.48–3.91)^**^			2.38	(1.45–3.90)**	2.34	(1.38–3.97)^*^
Not speaking language	0.56	(0.26–1.19)			0.65	(0.40–1.04)	0.97	(0.39–2.41)
No social support (practical)	2.12	(1.31–3.41)^*^			2.20	(1.24–3.28)^*^	1.79	(1.04–3.07)^*^

Unadjusted (OR) and adjusted Odds Ratios (AOR) and 95% confidence intervals (95%CI) of the association between predisposing and enabling variables on the outcome. Crude model: Model I: adjusted for sex, age, marital status, sexuality, education, and waiting time for residence permit ; Model II: adjusted for financial difficulties, social support, language skills; Model III: Fully adjusted for predisposing + enabling variables

^*^Significance level *p* < 0.05

^**^Significance level *p* < 0.001

### Estimation of the indirect effect of mediating variables

Bootstrap analyses were performed to assess some of the indirect pathways by estimating the proportion of the indirect effect (IE) on the total effect of the association (TE) that could be attributed to different mediating variables. The result showed that for unmarried persons, the partially mediating effect of the two risk factors, sexual coercion, and alcohol consumption, accounted for 13.1 and 15.9% respectively, of the total effect of the relationship between marital status and STI/HIV-testing. This pathway was also mediated by language skills, with an indirect effect accounting for 6.7% of the total effect of the association, indicating that being able to communicate in Swedish, compared to not, increased the probability of having had a STI/HIV test among unmarried persons. The other enabling factors seemed to have less influence on this pathway (IE = 1.4 and 0.06% of the total effect of the association, for social support and financial situation, respectively). The results showed that the pathway between sexual orientation and STI/HIV-testing was mediated by risk exposures. Among persons identifying as bi- or homosexual, experiences of sexual coercion accounted for 46.4% of the total effect of the association between sexual orientation and testing. The indirect effect of alcohol consumption and substance use was 10.8 and 13.9%, respectively. With regards to the “migrant specific” pathway, the results indicated that language skills and trust in health care had a protective mediating effect on the relationship between longer waiting time for residence permit and STI/HIV-testing and reduced the total effect of the association resulting from the exposure (IE = − 9.9% and − 3.4%, for language and trust in health care, respectively). That is, language skills and trust in health care reduced the probability of testing amongst those with longer waiting time. Further, the mediating effect of language on the pathway between education and STI/HIV testing was estimated to account for 27.8% of the total effect of the association, suggesting that almost one third of the relationship between higher levels of education on the probability of testing could be explained by language skills (Table 6).

**Table 6:** Odds Ratio (OR) indicating the total, direct, and indirect effect of the association between variables and STI/HIV-testing. MILSA 2.5 data on migrants from Syria and Iraq residing in Scania, Sweden who received residence permit between 2012 and 2016 (*n* = 3226)

	STI/HIV-Testing			
	Total Effect(TE) OR (95%CI)	Direct Effect(DE) OR (95%CI)	Indirect Effect (IE) OR (95%CI)	% Indirect Effect of total effect
**Pathway “Marital status”**				
IE *sexual coercion*	3.89 (2.59–5.84)	3.20 (2.11–4.86)	1.21 (1.08–1.36)	13.1%
IE *social support*	3.73 (2.39–5.82)	3.66 (2.38–5.63)	1.01 (0.96–1.07)	1.4%
IE *financial situation*	3.67 (2.42–5.55)	3.64 (2.40–5.50)	1.00 (0.98–1.03)	0.06%
IE *alcohol consumption*	3.74 (2.41–5.81)	3.01 (1.91–4.76)	1.23 (1.07–1.43)	15.9%
IE *language*	3.74 (2.49–5.62)	3.43 (2.25–5.21)	1.09 (1.03–1.15)	6.7%
**Pathway “Sexuality”**				
IE *sexual coercion*	1.84 (0.60–5.62)	1.37 (0.40–4.74)	1.33 (0.96–1.84)	46.4%
IE *alcohol consumption*	1.74 (0.46–6.53)	1.64 (0.45–5.94)	1.06 (0.92–1.22)	10.8%
IE *drug-use*	1.76 (0.61–5.06)	1.62 (0.55–4.75)	1.08 (0.89–1.31)	13.9%
IE *social support*	1.80 (0.55–5.81)	1.73 (0.54–5.50)	1.03 (0.95–1.12)	6.0%
**Pathway “Education”**				
IE *language*	2.08 (1.24–3.49)	1.69 (1.06–2.68)	1.23 (1.02–1.48)	28.7%
**Pathway “Waiting time”**^**c**^				
IE *trust in health care*	1.87 (0.93–3.76)	1.99 (0.98–4.03)	0.93 (0.87–1.00)	−3.4%
IE *language*	1.15 (0.73–1.80)	1.17 (0.75–1.84)	0.98 (0.95–1.01)	−9.9%

Odds Ratios (OR) and 95% confidence intervals (95%CI) for the Total Effect (TE), Direct effect (DE) and Indirect effect (IE) of the pathways between predisposing variables and the outcome, and % of the indirect effect of the mediating variable on the total effect of the association

^*^Significance level *p* < 0.05

## Discussion

This study aimed to explore general predictors for the uptake if STI/HIV-testing, as well as predictors specific to the migrant situation. To our knowledge, this is the first study that have investigated potential pathways to STI/HIV-testing among Syrian and Iraqi migrants who recently have resettled in Sweden. While the results from this study show similar associations to those found in other research in that younger age, bi-or homosexual, being unmarried, and higher education level predicted STI/HIV screening [20, 23, 27], it also contributes with novel knowledge about some of the pathways through which these factors operate, mediated by economic and social resources, language skills, and exposure to risks. The result deepens our understanding of processes that underlie the uptake of STI/HIV-testing that is crucial for designing targeted preventive activities and services. This is of importance for public health as the uptake of disease preventing and health promoting activities is generally lower among foreign-born compared to the general population [24, 31].

The result indicated that higher education predicted STI/HIV-testing directly, regardless of access to enabling resources or risk exposures. This pathway was partly mediated by access to social and economic resources, language skills and trust in health care. Language skills seemed to play a particularly important role. Research show that persons with higher levels of education are more likely to seek preventive health services because of their health knowledge and access to enabling resources [19]. Moreover, to refrain from seeking care due to discimination is more common among those with lower levels of education, and persons with mother tongue other than Swedish more often report poor encounters with the health care services [24]. Our result also suggested that the pathways from age, marital status, and sexuality to testing were indirect and operated via experiences of sexual coercion and alcohol consumption, indicating that utilization of STI/HIV-testing services was motivated by a perceived risk-awareness and sexual risk exposure. This is in line with other studies showing that risk awareness and known risk behaviours are associated with a greater likelihood of HIV-testing, while low perceived risk and absence of symptoms have been associated with a lower probability of testing and late presentation [20, 22]. Importantly, most HIV-cases in Sweden have been transmitted from individuals who were unaware of their infection [7]. Risk perception is largely based on correct knowledge about STI/HIV transmission [20, 22]. The strong relationship between the risk factors used as proxies for need and testing indicated that those at risk to a larger extent utilized services. This is promising as STI/HIV-testing is a critical entry point for accessing treatment, prevention of further transmission or risk behaviours [15, 16].

The prevalence of different forms of sexual exploitation among migrants in Europe is believed to be high [40]. The prevalence of experiences of sexual coercion in this study was 3% (2.6 and 3.3% for men and women, respectively), which is a low estimate. A possible explanation is underreporting due to sensitivity or differential perception of the concept. After adjusting for covariates, the result indicated that unmarried persons and bi- or homosexuals were two to three times more likely to have been exposed to sexual coercion. Further, sexual coercion was estimated to account for more than one tenth and almost half of the total effect of the relationship between marital status and sexuality and STI/HIV-testing, respectively. Thus, the lower probability of testing among those who did not have financial difficulties, or who did not lack access to social support, can be explained by the negative relationship with sexual coercion. That is, persons with lower access to social support and financial difficulties, were more likely to have experienced sexual coercion. Moreover, the proportion of the indirect effect of the association between substance use and alcohol consumption and testing among bi- or homosexuals was 13.9 and 10.8%, respectively, suggesting that bi- or homosexual may be exposed to multiple risks, as well as having lower access to enabling resources, compared to their heterosexual counterparts.

The asylum process is characterized by uncertainty, difficulties in building social networks, and mistrust of the immigration system and other institutions, influencing access to health care [41, 42], as well as increases risk exposure for STI/HIV [26]. Studies have highlighted language and supportive post-migration structures as critical factors in shaping the experiences of trust [41]. A prolonged asylum process can delay resettlement and negatively affect access to activities aiming to facilitate integration such as language training and civic- and health information [43]. This result indicated that longer, rather than shorter, waiting time for residence permit was associated with a greater likelihood of STI/HIV-testing. The mediating effect of language skills, and to a lower extent, trust in health care, on the relationship between waiting time and testing seemed to act in a way so it reduced the probability of testing. One possible explanation is that among those with longer asylum process, language skills and trust had a protective effect against other risk behaviours for STI/HIV. Too long time may have passed to be able to see an effect of waiting time on testing behaviour in this study. Further, the study participants were recruited after a two-year establishment phase, whereby migrants in Scania with residence permit get access to civic- and health system information, including information about sexual and reproductive health [34, 44, 45]. The relationship between the time of the asylum process, sexual risk behaviour, and the impact of integration-promoting activities, such as language training and civic- and health information, on access to STI/HIV prevention is a topic for future study. Securing access to preventive services and supportive living conditions is of great importance to achieve health equity. This study highlights the need for translated information about available testing facilities and where to turn for advice related to sexual health and rights, including psychosocial aspects of sexual relationships. It emphasizes a need to address structures and circumstances related to the asylum process and the post migration situation that increase people’s risk exposure to STI/HIV.

## Methodological considerations

Migrants are commonly underrepresented in population-based surveys, which contributes to an unstable basis for the development of preventive activities [46]. The questions in this survey were translated to the native language of the study participants, which have been shown to increase participation [47]. It was provided both on paper (response 31%) and online (response rate 69%) and piloted on representatives from the study population prior to the data collection. Almost all eligible candidates from the study population were invited to participate (up to the limit of 10,000). In total 3226 respondents returned the survey (32.3%). More men than women answered the survey (32.7 and 31.5%, respectively), more persons in the oldest age group compared to the younger (22.3% age group 22–29 and 44.3% age group 50–70), and more persons from Syria than Iraq answered the survey (35.4 and 18.8%, respectively). A larger part of the respondents represented the high-income category than low-income (41.2 and 29.0%, respectively). Skewness in the non-responses was managed by weighting the data according to population distribution of gender, age, birth country, income, and municipality [35, 48].

More than 5 % of the study participants responded “don’t remember” on the question if they had tested for an STI/HIV within the past 12 months. Recall of past utilization events are prone to inaccurate or incomplete recollection [49]. We decided to recode “don’t remember” as missing due to difficulties with interpretation. Similarly, it is common to exclude don’t know (DK) responses from the analysis [50]. Stigma and taboo are strong determinants of access to sexual health help seeking [22, 23]. This may also have affected the response to questions on sexual health and sexuality, which possibly explain the proportion of missing data for the questions on sexual orientation (18.8%) and sexual coercion (4.9%). Further, the perception and meaning of sexual coercion may differ for persons who originate from countries with patriarchal structures and traditional gender roles, including concepts of marital duty [51]. Another aspect to consider is that the survey was sent to respondent’s home address, thus response bias may be present as we cannot control the possible influence of family members on the answers [48].

Causal diagrams are beneficial for illustrating biases; however, they do not give an estimate of the magnitude of the bias. Including many covariates risk overadjustments and loss of power, particularly when stronger predictors are entered in the model. This may explain the loss of statistically significant association between enabling variables on the outcome in the third model [52]. Moreover, we used proxies for the “need” predictors in terms of sexual coercion and risk behaviours related to sexual risk taking, as data on actual need, such as symptoms, or known risk exposure, was unavailable. This limits our possibilities to draw conclusions on causal pathways. We cannot be sure if those who had not tested, had not done so because of a non-existing need, or whether it was due to barriers to access. The result from this study thus provides us with new knowledge about what influences the utilization of STI/HIV-testing services among those who have tested. A different study design is needed to assess causal pathways to testing. Importantly, migrants are a heterogenous group, and testing behaviours differ depending on the population and context studied [20, 22, 23]. Given the measurement issues considered above, these results may not be applicable beyond this study population and setting [48]. However, persons who are new to a country and unfamiliar with system and culture typically face similar barriers to accessing health care [19, 42]. These results also confirmed individual predictors of STI/HIV testing and sexual health help-seeking found in other studies [20, 22, 23].

## Conclusion

This study suggested that younger age, higher education, and identifying as bi-or homosexual predicted uptake of STI/HIV-testing among Syrian and Iraqi migrants who settled in Sweden. The pathways to testing were partially mediated by social and economic assets, host country language skills, and trust in health care. STI/HIV-testing also appeared to be largely motivated by sexual risk exposure and risk awareness. Language skills can act protectively against risks related to STI/HIV, as well as enhance access to testing. Interventions should focus on translated information, and to promote sexual health and rights from a broader integration perspective that address structures related to the asylum process and post-migration situation that increase people’s risk exposure to STI/HIV. Civil society organisations and outreach activities may have a particularly important role in reaching vulnerable subgroups of migrants. Qualitative or mixed method studies can complement with in-depth information about underlying factors that influence pathways to testing and identify specific needs that can inform a plan for action.

## Data Availability

The datasets generated during this study are not publicly available due to terms agreed upon the ethical approval but are available from the second author on reasonable request.

## Abbreviations

DAG: Directed acyclic graph
STI: Sexually Transmitted Infection
HIV: Human Immunodeficiency Virus
SRHR: Sexual and Reproductive Health and Rights
EU: European Union
HSU: Health Service Utilization
MENA: Middle East and North Africa regions
NGO: Non- Governmental Organisation
MSM: Men who have Sex with Men
